# Exploring Mesenchymal Stromal Cells as a Potential Therapy for Comorbid Osteoarthritis and Type 2 Diabetes Mellitus

**DOI:** 10.1155/sci/2681413

**Published:** 2025-12-13

**Authors:** Meiling Liu, Siyi Xie, Yajie Kong, Yiming Yang, Ruixue Chen, Yuzhong Wang, Shuxing Cao, Yongzhou Song

**Affiliations:** ^1^ Department of Orthopedics, The Second Hospital of Hebei Medical University, Shijiazhuang, Hebei Province, China, hebmu.edu.cn; ^2^ Department of Hebei Medical University-National University of Ireland Galway Stem Cell Research Center, Hebei Medical University, Shijiazhuang, Hebei Province, China, hebmu.edu.cn; ^3^ Hebei Technology Innovation Center for Stem Cell and Regenerative Medicine, Shijiazhuang, Hebei Province, China

**Keywords:** diabetic osteoarthritis, inflammation, mesenchymal stromal cells, osteoarthritis, type 2 diabetes mellitus

## Abstract

**Background:**

In recent years, the incidence rates of type 2 diabetes mellitus (T2DM) and osteoarthritis (OA) have increased significantly. Currently developed therapeutic approaches (e.g., pharmacotherapy) exhibit limited efficacy in the treatment of T2DM and OA, failing to fully restore joint function and pancreatic islet function. Mesenchymal stem cells (MSCs) have demonstrated substantial potential in repairing cartilage damage, reducing blood glucose levels, and other related aspects.

**Objective:**

This review aims to evaluate whether MSC therapy represents a potential therapeutic strategy for T2DM complicated with OA.

**Summary:**

This review highlights the association between OA and T2DM and summarizes the applications of MSCs in the treatment of OA and T2DM, the potential mechanisms of MSCs, as well as relevant therapeutic strategies.

**Conclusion:**

MSC therapy may exert therapeutic effects in models of T2DM complicated with OA. This approach is expected to serve as an innovative and effective therapeutic method, promoting research on stromal cells and their applications. However, further studies are still required to verify its safety and feasibility.

## 1. Introduction

**Figure 1:**
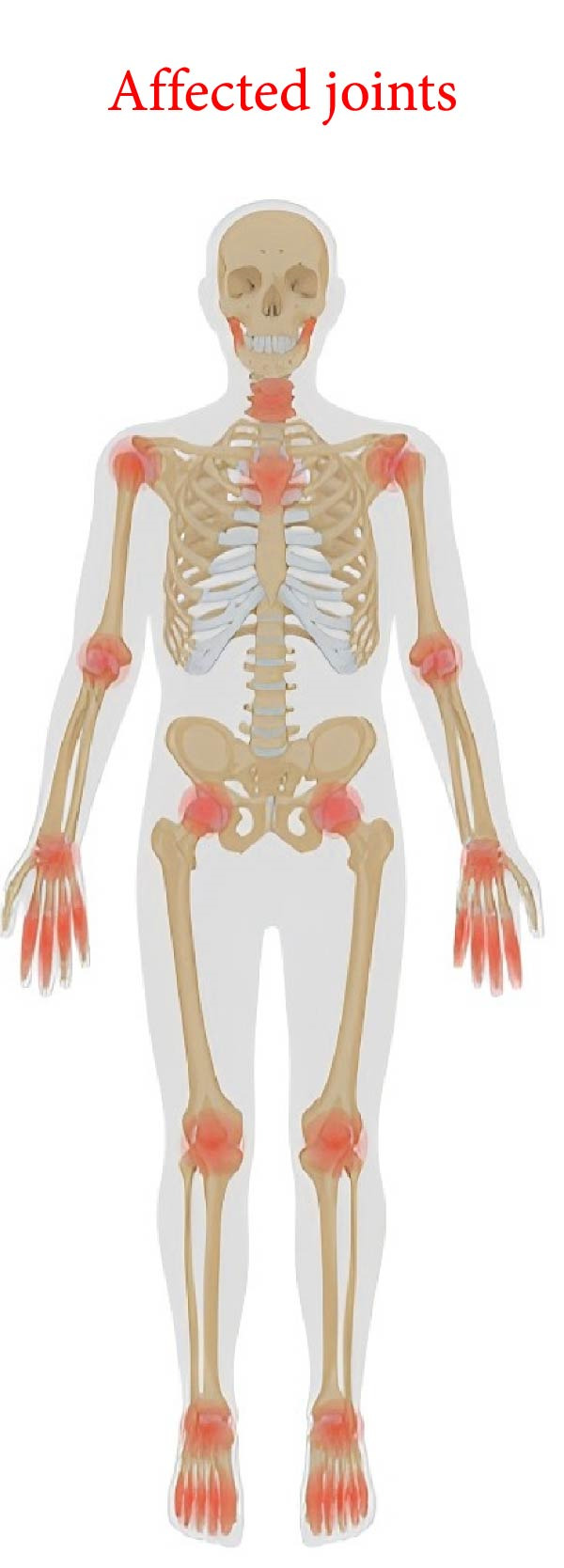
Affected joints. Image created with BioRender (www.biorender.com).

Osteoarthritis (OA) is a prevalent, incapacitating, and intricate chronic disorder frequently associated with multiple diseases. OA is a total joint syndrome that encompasses structural alterations in the articular cartilage, subchondral bone, ligaments, joint capsules, synovium, and muscles surrounding the joint (Figure 1) [1]. The pathological alterations of OA are multifaceted, encompassing synovitis, cartilage degeneration, subchondral bone thickening, osteophyte formation, ligament degeneration, meniscus injury, and changes in the surrounding muscle structure [2]. The primary feature of OA is the inability of the damaged cartilage to undergo the repair process adequately, attributed to biomechanical and biochemical alterations in the affected joints [3]. In addition, age and gender are significant determinants of OA [4].

Type 2 diabetes mellitus (T2DM) is a metabolic disorder arising from the lack of insulin cell response (insulin resistance), consequently resulting in subsequent hyperglycemia [5]. The intricate interplay of *β*‐cell dysfunction and insulin resistance is the fundamental characteristic of the complexity of T2DM [6].

T2DM is an established risk factor for multiple comorbidities, such as cardiovascular disease and osteoporosis [7–9]. Elevated bone fragility and increased fracture risk are common complications among patients with long‐term T2DM [10]. Because T2DM is a major metabolic disorder, it has a negative impact on cartilage and joints due to oxidative stress, pro‐inflammatory cytokines, chronic high glucose concentration, and insulin resistance [11].

**Figure 2:**
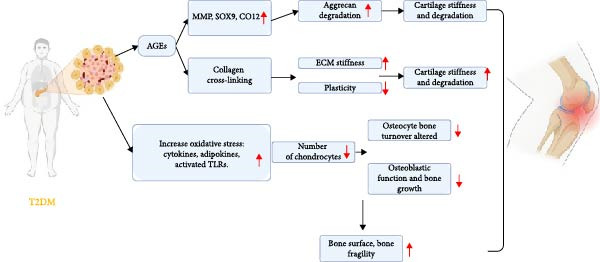
The diagram provides an overview of how T2DM affects OA. Image created with BioRender (www.biorender.com).

An increasing number of researchers have started looking into the connection between T2DM and OA after it was found that T2DM can either hasten or contribute to the development of OA (Figure 2) [12]. They coexist intermittently due to shared common risk factors such as obesity and aging, leading to a higher prevalence [2].

Dubey et al. [13] also carried out relevant statistical experiments and discovered that the histopathology and western blot studies of the knee joints of diabetic mice exhibited decreased levels of advanced glycation end products, matrix metalloproteinase‐1, and chondro‐specific proteins. The findings imply that DM is strongly associated with knee OA (KOA), and obesity might not be a confounding factor. This one is strongly associated with eliminating other confounding factors, further verifying that T2DM is one of the risk factors for OA.

Currently, traditional treatment approaches for OA are mainly categorized into two types: pharmacological intervention and surgical treatment [14]. Pharmacological treatment centers on anti‐inflammation and inhibition of catabolism as its core functional directions [15]. Surgical treatment focuses on the repair of local cartilage damage. In the clinical treatment regimens for T2DM, hypoglycemic drugs and insulin therapy are the mainstream options [16]. However, there is still a lack of methods that can achieve a radical cure for the disease. Although pharmacological and other adjuvant treatments can make patients’ blood glucose levels approach the normal range as much as possible [17], thereby delaying or even preventing the occurrence of diabetes‐related complications [18]. All existing treatment regimens can alleviate symptoms and reduce patients’ physical discomfort to a certain extent; unfortunately, none of these methods can achieve the effect of completely curing the disease.

The potential application value of mesenchymal stem cells (MSCs) in the treatment of OA and T2DM has been confirmed by numerous studies [19]. Specifically, MSCs can not only facilitate the repair and regeneration of damaged cartilage tissue by regulating the proliferation and differentiation of chondrocytes and promoting the synthesis of extracellular matrix [20, 21], thereby alleviating the progression of cartilage damage in the pathological process of OA, but also reduce both OA‐related joint inflammation and the systemic low‐grade inflammatory response associated with T2DM through mechanisms such as regulating the secretion of inflammatory factors and inhibiting the activation of inflammatory cells [22, 23]. These properties provide a new direction for the coordinated intervention of the two diseases.

Therefore, focusing on the treatment of diabetes‐related OA with MSCs, this paper, by integrating mechanistic and clinical studies, sorts out the current application status of MSCs, analyzes their feasibility and therapeutic efficacy, summarizes the latest research progress, and provides directions for subsequent studies.

## 2. Pathways Linking T2DM to OA: Hyperglycemia, Inflammation, and AGEs

Recent research has demonstrated that T2DM significantly contributes to the pathology of OA through two primary pathways. First, persistent hyperglycemia resulting from T2DM promotes oxidative stress and subsequent production of pro‐inflammatory cytokines and AGEs in the joint tissues [24, 25]. This exacerbates inflammation and damages the affected joints, and the effects of insulin resistance, which occurs in patients with T2DM, can occur both locally and through low‐grade inflammation throughout the body. Accumulation of cytokines such as IL‐1, IL‐6, and TNF *α*, ultimately leading to OA occurring [25].

Through the first pathway, chondrocytes are capable of detecting changes in extracellular glucose levels and adjusting themselves by regulating GLUT‐1 synthesis and lysosomal‐mediated degradation [26]. However, due to chondrocyte damage in patients with OA, impaired downregulation of GLUT‐1 leads to intracellular glucose accumulation [26]. Chondrocytes in the setting of OA are unable to adapt to elevated extracellular glucose concentrations, resulting in the build‐up of AGEs and the expression of inflammatory and catabolic mediators, including pro‐inflammatory cytokines and matrix metalloproteinases [27].

The accumulation of AGEs in cartilage results in increased brittleness, as well as the induction of pro‐inflammatory and procatabolic states in chondrocytes [28]. Additionally, AGEs have been demonstrated to downregulate AMPK*α*/SIRT1/PGC‐1*α* signaling in chondrocytes, leading to oxidative stress, heightened inflammation, and elevated apoptosis that ultimately contributes to mitochondrial dysfunction [29].

The second pathway leading to T2DM is characterized by visceral obesity, which triggers chronic low‐grade systemic inflammation and subsequently results in joint metabolic disorders and OA [30]. Chondrocytes express insulin receptors, rendering these cells sensitive to insulin. Although insulin normally suppresses synovial inflammation and catabolism, obese individuals with T2DM experience synovial inflammation that diminishes the inhibitory effect of elevated insulin levels on OA‐induced inflammation and catabolic mediators [31].

## 3. The Influence of T2DM on Articular Cartilage

**Figure 3:**
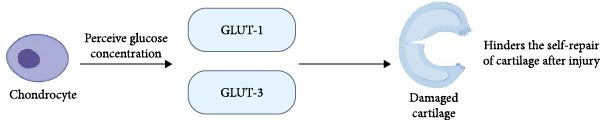
The effect of a high‐glucose environment on cartilage. Image created with BioRender (www.biorender.com).

Articular cartilage is a nonvascularized and noninnervated tissue that receives nutrients through the connection of the joint cavity with the subchondral bone and synovial fluid [32]. Articular cartilage is composed of an extracellular matrix, and the extracellular matrix contains one type of cell: chondrocytes. The matrix mainly consists of water, proteoglycans, and type 2 collagen [1]. Due to the catabolic activity of chondrocytes, the turnover rate of this matrix component is low. Chondrocytes are glycolytic cells that express glucose transporter proteins (GLUTs), especially GLUT‐1 and GLUT‐3, capable of sensing glucose concentration in the culture medium and adapting GLUT expression and membrane incorporation (Figure 3) [33]. The absence of blood vessels and nerves hinders self‐repair after cartilage injury [34].

Wan et al. [35] carried out microindentation tests to determine the mechanical properties of cartilage in healthy rats and diabetic rats and found that T2DM notably modified the cartilage. Wang et al. [36] further carried out a preclinical experiment in this regard. They found that the articular cartilage degeneration and aging in the elderly T2DM–induced OA group were more severe than those in the other groups. A high glucose concentration accelerates the degradation and aging process of cartilage.

Jain et al. [37] investigated the role of hyperglycemia in the context of T2DM and OA. The results showed that under oxygenated conditions, OA chondrocytes increased glucose consumption and the production of catabolic enzymes (MMP13 and ADAMTS5) in response to elevated extracellular glucose levels, while chondrocytes from normal cartilage did not exhibit this response [37]. This study confirmed that hyperglycemia can promote chondrocyte‐mediated OA cartilage degradation.

As demonstrated in the study by Tsai et al. [38], when chondrogenesis was induced, the chondrogenic potential of human mesenchymal stem cells (hMSCs) maintained under high glucose conditions was lower than that of cells maintained under low glucose conditions. Therefore, a further conclusion was drawn that hMSCs expanded in high‐glucose cultures exhibit reduced chondrogenic potential, indicating that glucose concentration influences the regulation of chondrogenic capacity in predifferentiated hMSCs.

## 4. The Influence of T2DM on Subchondral Bone

**Figure 4:**
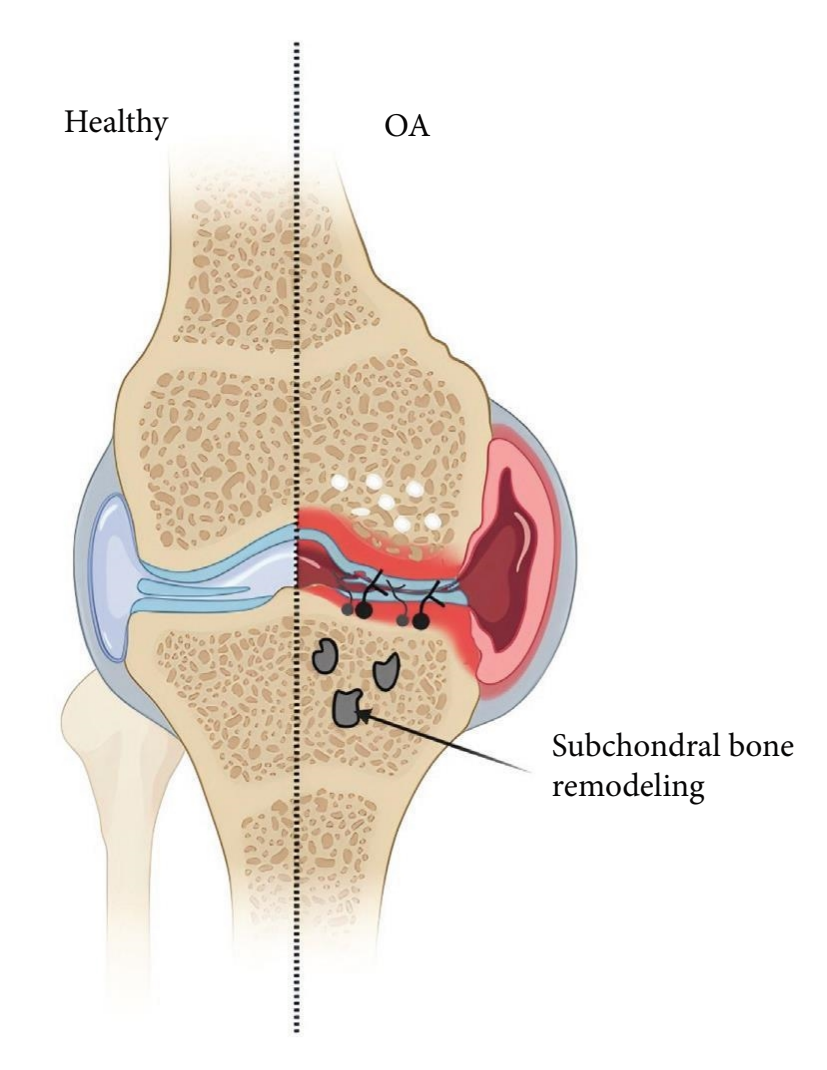
Healthy subchondral bone (on left) vs. subchondral bone with OA (subchondral bone undergoing remodeling shown). Image created with BioRender (www.biorender.com).

The subchondral bone (Figure 4) lies between the articular cartilage and the bone tissue and consists of the subchondral bone plate in the deep epiphyseal area of the cartilage and the underlying trabecular bone [39]. The close integration of cartilage and subchondral bone forms a load‐bearing structure that maintains a dynamic equilibrium. Its principal functions are to absorb the gravitational load endured by the joint and transfer and distribute it to other places. Additionally, it can maintain the dynamic balance among the various structures within the joint by modulating metabolism and adjusting the shape of the joint [39].

Studies have shown that T2DM can also cause a certain degree of damage to the subchondral bone. Wen et al. [40] found that 28 patients with hypertension, T2DM, and KOA had significant bone loss in the subchondral plate and lower bone mineral density (BMD). It can be seen that there is a biological association between the bone loss of the KOA subchondral plate and hypertension as well as T2DM.

Gui et al. [41] established a rat model of T2DM using streptozotocin combined with a high‐sugar and high‐fat diet and found that T2DM affects subchondral bone remodeling primarily by inhibiting bone formation. Another study also revealed that in areas where the cartilage remained intact, the underlying bone in the diabetic group still exhibited abnormal remodeling [42]. This further confirms that T2DM can cause damage to the subchondral bone.

## 5. The Influence of T2DM on the Synovium

The synovial tissue, which is the primary cell type responsible for generating synovial fluid in joints, plays a pivotal role in the metabolism and material exchange of cartilage [43]. Given the relatively poor blood supply to the articular cartilage and the abundant vasculature in the synovial tissue, the synovium might be more prone to high glucose stimulation [44].

Ribeiro et al. [45] found in an animal model that the DM group exhibited more synovial inflammation than the non‐DM group. In addition, Luo et al. [46] also found that the expression of MMPs in the synovial fluid of the DM‐OA group was significantly higher than that in the OA group and the healthy control group [45, 46]. All these indicate that T2DM also exerts a significant impact on the synovium.

## 6. Comparison Between Traditional Therapies and MSC Therapy

Traditional therapeutic regimens often have the limitation of “single intervention,” and thus, struggle to meet the multidimensional needs of the disease [47]. Specifically, although traditional anti‐inflammatory drugs can act on the relief of OA symptoms, their long‐term therapeutic effects have not yet been supported by clear clinical evidence [48], and adverse reactions may occur during medication [49]. Insulin therapy, while controlling blood glucose [50], carries the risk of inducing hypoglycemia. Oral hypoglycemic drugs may cause problems such as weight fluctuation and gastrointestinal discomfort [51], and their efficacy needs to be maintained through regular administration, which imposes high requirements on patient compliance.

MSCs have demonstrated potential application value in the treatment of OA and T2DM. In the treatment of OA, these cells can promote the repair and regeneration of cartilage tissue, reduce cartilage damage caused by arthritis [52], and alleviate inflammatory responses associated with both arthritis and diabetes, thereby improving joint pain symptoms and joint function in patients. However, their therapeutic efficacy still needs to be verified through larger‐sample clinical trials with control groups [53]. In terms of T2DM treatment, the application of MSCs shows good tolerability. Although some patients may experience hypoglycemia, it is also accompanied by symptoms such as nausea, vomiting, and headache [54]. The overall therapeutic effect presents a positive and promising trend based on a comprehensive analysis of existing research results.

## 7. MSC

Mesenchymal stromal cells (MSCs), multifunctional stromal cells, were initially identified in the bone marrow and can subsequently be harvested from tissues such as adipose, umbilical cord, placenta, and synovium [55]. The MSCS exhibited the expression of surface markers CD73, CD90, and CD105, while lacking the expression of hematopoietic markers CD45, CD34, CD14, and CD79 [56]. MSC possesses the capacity for self‐renewal and multidirectional differentiation potential and is capable of differentiating into adipocytes, osteoblasts, and chondrocytes [57].

The mechanism of action of MSCs on OA primarily relies on their multifaceted differentiation function, paracrine signaling, anti‐inflammatory immune modulation, and homing capability [58].

1. MSCs have the ability to migrate from subchondral bone to injured areas and undergo differentiation into chondrocytes and osteoblasts, contributing to the repair of cartilage and subchondral bone tissue [58].
2. Paracrine signaling is the primary mechanism through which MSCs contribute to tissue repair, as they mitigate tissue damage and facilitate tissue regeneration by releasing exosomes and a variety of bioactive factors with distinct properties [59].
3. Anti‐inflammatory and immune modulation to decrease the levels of chemokines and inflammatory mediators [60].
4. MSCs exhibit a preference for homing to sites of tissue injury and inflammation [61].

The primary mechanism of action of MSC on T2DM involves transdifferentiation into pancreatic secretory cells in vitro [62]. Additionally, MSCs facilitate the regeneration of endogenous islet *β* cells by migrating to injured islet cells [62].

However, relevant studies have shown that the diabetic microenvironment exerts an adverse effect on the regenerative capacity of MSCs. Specifically, this microenvironment not only induces phenotypic changes in MSCs but also impairs multiple of their key functions—including adipogenic differentiation, osteogenic differentiation, and angiogenic differentiation processes [63].

Keats and Khan [64] demonstrated that the activity of alkaline phosphatase (ALP)—a marker associated with osteogenic differentiation potential—was reduced in both dedifferentiated adipose‐derived stem cells (dADSCs) and adipose‐derived stem cells (ADSCs) treated with high‐glucose medium, which consequently impaired their osteogenic capacity. Zhang et al. [65] proposed that the decline in osteogenic capacity might also be associated with the upregulation of DNA methylation levels in glycosylated ADSCs induced by AGEs. Rennert et al.’s [65] study demonstrated that, in a high‐glucose microenvironment, the expression levels of cytokines (e.g., vascular endothelial growth factor [VEGF]) and their related receptors secreted by ADSCs were reduced. Additionally, the proangiogenic capacity of these ADSCs was weaker compared to normal ADSCs [65].

Meanwhile, the immunomodulatory properties and angiogenic potential of MSCs are also damaged as the duration of exposure to this microenvironment increases, and this series of effects still awaits further verification through subsequent experiments.

However, numerous preclinical and clinical trials of MSCs for T2DM and OA have demonstrated promising results, significantly mitigating the progression of these diseases. MSCs have shown potential in reducing inflammation, promoting tissue regeneration, and modulating the immune system, making them a promising therapeutic option for these chronic conditions [66].

## 8. MSC and OA

Numerous clinical trials have been conducted to investigate MSC and OA (Table 1).

**Table 1:** Summary of clinical trials related to OA and MSCs.

Author	Year	Participants	Formulations	Method of delivery	Results
Song et al. [67]	2018	18	Autologous haMSCs	Intra‐articular injection	The highest level of improvement was seen in the high‐dose group receiving 5 × 10^7^ cells
Emadedin et al. [68]	2018	43	Autologous MSCs	Intra‐articular injection	MSCs provided significant and clinically relevant pain relief within 6 months
Khalifeh Soltani et al. [69]	2019	20	Allogeneic placental MSCs	Intra‐articular injection	The cartilage thickness of the entire knee joint area improved in ~10% of the patients in the experimental group within 24 weeks
Lee et al. [70]	2019	24	AD‐MSCs	Intra‐articular injection	Patient feedback indicated that intra‐articular injection of autologous AD‐MSCs led to improved functionality and pain relief for knee OA patients
Chahal et al. [71]	2019	12	BM‐MSCs	Intra‐articular injection	The highest cellular dose of 50 × 10^6^ BM‐MSCs showing potentially greater effectiveness
Dilogo et al. [72]	2020	29	hUC‐MSC	Intra‐articular injection	The study results indicate that the maximum effect of hUC‐MSCs was achieved 6 months after injection
Lamo‐Espinosa et al. [73]	2020	60	BM‐MSCs + PRGF	Intra‐articular injection	The study found that the OA symptoms of these patients significantly improved
Sadri et al. [74]	2022	3	AD‐MSCs	Intra‐articular injection	The intra‐articular injection of AD‐MSC was safe and may be effective for cartilage regeneration in knee OA
Sadri et al. [75]	2023	40	Allogeneic AD‐MSCs	Intra‐articular injection	100 × 10^6^ allogeneic AD‐MSCs can alleviate KOA complications through inflammatory regulation and induction of cartilage regeneration
Chen et al. [76]	2024	11	AD‐MSCs	Intra‐articular injection	The results indicated that the 4 × 10^7^ dose of AD‐MSCs is more pronounced for joint repair

*Note:* AD‐MSCs, adipose‐mesenchymal derived stem cells; BMSCs, human bone marrow mesenchymal stem cells.

Abbreviation: UC‐MSCs, umbilical cord mesenchymal stem cells.

Song et al. [67] investigated the safety and therapeutic potential of autologous human adipose‐derived mesenchymal stem cells (haMSCs) in patients with OA. The study concluded that the high‐dose group, which received an intra‐articular injection of 5 × 10^7^ haMSCs, was safe and effective in relieving knee pain, enhancing function, and increasing cartilage volume.

Emadedin et al. [68] conducted a placebo‐controlled Phase 1/2 trial. The trial results showed that, compared with the placebo, mesenchymal stem cell implantation significantly relieved clinically relevant pain within 6 months. Although the follow‐up period was short and the sample size was small, it confirmed to a certain extent that MSCs are a potential therapeutic approach for the effective treatment of OA.

Khalifeh Soltani et al. [69] found that a single intra‐articular injection of allogeneic placental MSCs for the treatment of KOA is safe and can lead to clinical improvements after a 24‐week follow‐up period. In a study conducted by Lee et al. [70], patient feedback after injection treatment showed that intra‐articular injection of autologous AD‐MSCs improved function and relieved pain in patients with KOA, without causing adverse events during the 6‐month follow‐up period.

Chahal et al. [71] conducted an experiment with 12 patients, administering different doses of BM‐MSCs for injection treatment, including 1 × 10^6^, 10 × 10^6^, or 50 × 10^6^ cells. It was observed that all treated patients experienced varying degrees of relief, with the highest cellular dose of 50 × 10^6^ BM‐MSCs showing potentially greater effectiveness [71]. It is evident that the improvement of the symptoms of these patients confirmed the effectiveness and safety of MSC in the treatment of OA.

Dilogo et al. [72] recruited 29 subjects, dividing them into mild and severe groups. They injected the subjects with 10 × 10^6^ units of hUC‐MSC and conducted an observation study over the period of 1 year [72]. The study results indicate that the maximum effect of hUC‐MSCs was achieved 6 months after injection. This clinical trial may be revisited to explore the most suitable treatment course.

Lamo‐Espinosa et al. [73] conducted a study to confirm the optimal dose and the effectiveness of adjuvants in the treatment for KOA. They selected 100 × 10^6^ BM‐MSCs combined with platelet‐rich plasma (PRGF) as an adjuvant and observed its clinical effect on 60 patients diagnosed with KOA [73]. The patients were randomly assigned to receive intra‐articular administration of 100 × 10^6^ BM‐MSCs combined with platelet‐rich plasma (PRGF) three times a week. The study found that the OA symptoms of these patients significantly improved. The combined treatment of MSC and PRP showed promise, but further support from phase III clinical trials is still needed.

Sadri et al. [74] recruited three patients with KOA and intra‐articularly injected a total of 100 × 10^6^ AD‐MSCs to observe whether AD‐MSCs are also highly effective. They conducted a 6‐month follow‐up, and the results showed that the intra‐articular injection of AD‐MSC was safe and may be effective for cartilage regeneration in KOA. However, more individuals need to be observed, and a BM‐MSCs group should also be added for comparison to further determine which one is more effective.

In the past 2 years, researchers started conducting additional experiments, including Phase 3 trials, to compare the effectiveness of different sources of MSC. So a study by Pintore et al. [77] compared the effectiveness of two different sources of MSCs. They conducted experiments on 51 patients who received a particular type of stem cell injection (BMAC) and 51 patients who received a different type (ADSC). The results, after a 6‐month follow‐up, showed that both types of injections significantly improved pain and functional outcomes in patients with KOA. However, there was no statistically significant difference in clinical and functional outcomes between the two groups. Further high‐quality clinical trials on a larger scale are needed to confirm these findings.

Kim et al. [78] conducted further research on AD‐MSC again because they believed that the previous studies on AD‐MSC were not detailed enough, and the small sample size was insufficient to demonstrate the effectiveness of AD‐MSC in the treatment of OA. Therefore, they carried out a Phase 3 multicenter clinical trial, it was finally obtained that intra‐articular injection of autologous cultured and expanded ADMSC can significantly relieve pain and improve functional in patients with K–L Grade 3 OA. However, magnetic resonance imaging showed no significant difference in cartilage defect changes between the two groups at 6 months [78]. Therefore, whether AD‐MSC ultimately has a good effect or not requires longer follow‐up and evaluation.

To further address the shortcomings observed in the previous AD‐MSC experiments, Sadri et al. [75] prolonged the treatment and follow‐up evaluation period for this purpose. The evaluation conducted 1 year later demonstrated that received intra‐articular transplantation of 100 × 10^6^ allogeneic AD‐MSCs can alleviate KOA complications through inflammatory regulation and induction of cartilage regeneration Bahareh. Administration of allogeneic AD‐MSCs is effective and brings improvements in clinical signs and symptoms.

While Chen et al. [76] explored to optimize which dose of AD‐MSCs was the best to test two dose levels: 6.7 × 10^6^ (low dose) and 4 × 10^7^ (high dose). The results indicated that the 4 × 10^7^ dose of AD‐MSCs is more pronounced for joint repair. This research again determined the optimal dose and provided a promising path for the treatment of OA with AD‐MSCs.

Currently, although MSCs still have limitations in the field of OA treatment—such as small sample sizes in studies, relatively short follow‐up observation periods, and the possibility of relevant side effects (including fever and vomiting) in some patients—their overall therapeutic efficacy shows a positive developmental trend based on existing research results. This provides an important option for the exploration of OA treatment and even cure strategies in the future.

## 9. MSC and T2DM

At present, multiple clinical experiments on MSC and T2DM have been conducted and are ongoing or have already concluded (Table 2).

**Table 2:** Summary of clinical trials related to T2DM and MSCs.

Author	Year	Participants	Formulations	Method of delivery	Results
Lu et al. [79]	2019	41	BM‐MSC	Local injection	BMMSC treatment can prolong limb salvage time and improve limb blood flow
Nguyen et al. [80]	2021	30	Autologous BM‐MSC	Intravenous and pancreatic artery injection	The data demonstrated that the administration of autologous BM‐MSC via the intravenous or dorsal pancreatic artery route is effective for the treatment of T2DM
Zhang et al. [81]	2022	14	hUC‐MSCs	Locally and intravenous injection	The preliminary study demonstrated the initial clinical benefits of hUC‐MSC for the healing of DFU
Zang et al. [82]	2022	37	UC‐MSCs	Intravenous injection	These results suggest that intravenous infusion of UC‐MSCs is an effective approach for improving T2DM
Lian et al. [83]	2022	16	hUC‐MSCs	Intravenous injection	The outcomes of this study suggest that the infusion of hUC‐MSC can ameliorate blood glucose, restore islet *β*‐cell function, and reduce the dosage of hypoglycemic drugs without inducing serious adverse events
Lian et al. [84]	2023	34	hUC‐MSCs	Intravenous injection	The study demonstrated that hUC‐MSC transplantation exhibits good tolerance and high safety in the treatment of T2DM

*Note:* AD‐MSCs, adipose‐mesenchymal derived stem cells; BMSCs, human bone marrow mesenchymal stem cells.

Abbreviations: DFU, diabetic foot ulcer; UC‐MSCs, umbilical cord mesenchymal stem cells.

First, Lu et al. [79] compared which of the bone marrow‐derived mesenchymal stem cells (BM‐MSCs) and bone marrow‐derived mononuclear cells (BMMNCs) is more suitable for treating diabetic complications. The results of this trial showed that, compared with BMMNC treatment, BM‐MSC treatment can prolong limb salvage time and improve limb blood flow. Furthermore, compared with conventional treatment, it can promote limb blood flow and ulcer healing and reduce ulcer recurrence. The publication of this study confirms the efficacy of BM‐MSC treatment for T2DM.

T2DM patients are prone to diverse complications. Whether MSCs can treat T2DM and its complications is an issue worthy of attention. Chen et al. [85] included a DM patient with recurrent lower extremity bullous DM in this study, local injection with a dose of 7.8 × 10^7^ BM‐MSC, and after 9 months of treatment. During the subsequent 10‐year follow‐up, this patient did not relapse. Autologous BMMSC transplantation therapy may be an effective approach for recurrent diabetic bullous; however, this study requires further investigation with an expanded sample size to reach a definite conclusion.

First, Zhang et al. [81] expanded the recruitment scope and enrolled 14 patients with peripheral arterial disease (PAD) and nonhealing diabetic foot ulcers (DFUs) to evaluate the safety and efficacy of human umbilical cord mesenchymal stem cell (hUC‐MSC) administration based on conservative treatment. This preliminary study demonstrated the initial clinical benefits of hUC‐MSCs for DFU healing. However, due to the still insufficient sample size, subsequent experiments involving a larger population are required.

BM‐MSCs are the most extensively tested cell type and are considered safe and effective in the treatment of T2DM. Nguyen et al. [80] were interested in the safety of administering BM‐MSCs to T2DM patients via intravenous injection versus pancreatic arterial injection. The data indicated that the administration of autologous BM‐MSCs through either the intravenous or dorsal pancreatic arterial route is effective for the treatment of T2DM.

Zang et al. [86] aimed to further determine the efficacy and safety of UC‐MSCs in Chinese adult patients with T2DM. They ultimately concluded that UC‐MSC transplantation is a therapeutic approach for Chinese adult T2DM patients.

To further evaluate the therapeutic effect of UC‐MSCs, Zang et al. [82] conducted a Phase 2 study. The study used UC‐MSCs at the 4th passage, with a total number of 1 × 10^6^ UC‐MSCs per kilogram administered in each infusion. Eventually, compared with the placebo group, the UC‐MSC group showed more significant differences in glycated hemoglobin (HbA1c) relative to the baseline at 9 and 48 weeks. These results indicate that intravenous infusion of UC‐MSCs is an effective method for improving T2DM.

Lian et al. [83] further evaluated the efficacy and safety of hUC‐MSC infusion for the treatment of T2DM. Efficacy was assessed by measuring fasting blood glucose, C‐peptide, normal glycated HbA1c, insulin resistance index, and pancreatic islet *β*‐cell function. The results of this study showed that hUC‐MSC infusion can improve blood glucose levels, restore pancreatic islet *β*‐cell function, and reduce the dosage of hypoglycemic drugs without inducing serious adverse events. Therefore, it further confirms that hUC‐MSC infusion is a viable option for the treatment of T2DM.

Lian et al. [84] also evaluated the safety of hUC‐MSC infusion for the treatment of T2DM. Finally, the study showed that hUC‐MSC transplantation exhibits good tolerability and high safety in the treatment of T2DM. It can enhance human immunity and inhibit lymphocytes.

Currently, although MSCs have not yet overcome all bottlenecks in the field of T2DM treatment—such as the limited sample size of most clinical studies, relatively short follow‐up observation periods, and the potential occurrence of treatment‐related adverse reactions in a small number of patients—comprehensive analysis of existing research data shows that their overall therapeutic efficacy presents a positive and promising development trend. This research progress provides a highly valuable new direction and alternative approach for the subsequent optimization of T2DM treatment regimens and even the exploration of radical cure strategies.

## 10. The Clinical Translation Prospects of Mesenchymal Stem Cell Therapy

MSCs, as a form of cellular therapy, have been the subject of extensive research. They exhibit promising application potential in areas such as tissue repair and regeneration and antitumor therapy. Unlike other cellular therapeutic approaches, the therapeutic efficacy of MSCs does not solely rely on cell–cell contact; it may also involve the so‐called “hit‐and‐run” mechanism [87].

To date, a number of mesenchymal stem cell therapies have been approved for use worldwide [88]. Recent research data indicates that encapsulating MSCs with biomaterials can enhance the cells’ retention rate and viability in in vitro environments [89]. However, further research and verification are still required to assess their functional effects in in vivo settings. MSC therapy still confronts substantial challenges. Continued exploration of diverse processing methods to address these hurdles can expand its clinical indications and enhance its therapeutic efficacy in treating T2DM complicated with OA.

## 11. Common Pathological Signaling Pathways and Shared Genes of T2DM–OA

**Figure 5:**
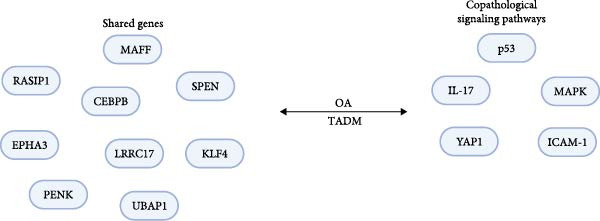
Copathological signaling pathways and shared genes of T2DM–OA. Image created with BioRender (www.biorender.com).

Given the high prevalence rates as well as common risk factors of OA and T2DM, their coexistence is frequent. The aim is to gain a more lucid understanding of the common pathological signaling pathways and biomarkers between diabetes and OA, with the aspiration to address the issues induced by these two disorders. With an escalating number of scholars uncovering the inextricable connection between T2DM and OA, Song and Yu [90] probed into the shared genes between T2DM and OA and identified 12 shared genes (Figure 5) [90–92]. These genes collectively participate in several key signaling pathways, such as the p53, IL‐17, NF‐kB, and MAPK signaling pathways (Figure 5) [90, 93].

**Figure 6:**
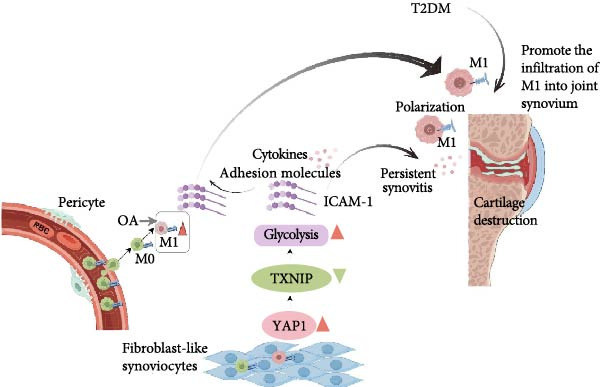
Association between YAP1/TXNIP signaling axis and metabolic OA. Image created with Figdraw (www.Figdraw.com).

The latest research by Yang et al. [94] found OA will produce more macrophages 1 (M1), which is more M1 accumulation, aggravating inflammation. However, metabolic diseases such as T2DM will promote the infiltration of macrophages into the joint synovium. In addition, T2DM will also promote the increase of ICAM‐1 and other levels, which will further aggravate the progression of inflammation [94]. They further revealed the role and mechanism of the Hippo‐yes‐associated protein 1 (YAP1) and ICAM‐1 signaling pathways in the pathogenesis of metabolic OA (Figure 6).

The YAP1/TXNIP signaling axis will increase glycolysis, promote the increase of ICAM‐1 and other cytokines, and further promote the recruitment of macrophages into the joint, thus, spreading inflammation and aggravating OA [95]. Targeting YAP1 is a promising approach for alleviating metabolic OA diseases. The role and mechanism of YAP1 signaling in regulating the expression and translocation of the key glycolytic transporter GLUT1 are conducive to further exploring the mechanism of synovial energy metabolism disorder involved in inflammatory and metabolic joint diseases [94]. Targeting YAP1 signaling to regulate synovial metabolic reprogramming is expected to provide new ideas for the diagnosis and treatment of T2DM andOA [94].

Dong et al. [96] identified MFAP5 as the most significantly upregulated extracellular matrix‐associated gene in the infrapatellar fat pad (IPFP) of diabetic mice. Furthermore, high glucose levels significantly induce the expression of MFAP5 and targeting MFAP5 attenuates fibrosis and inflammation in the IPFP of DM mice. These findings provide compelling evidence for a critical role of MFAP5 in the development of metabolic OA.

## 12. Conclusion

This article systematically summarizes the research progress of MSCs in the treatment of T2DM and OA. The results show that although the current technical systems and clinical applications of mesenchymal stem cell therapy for these two diseases are not yet mature, existing studies have limitations such as small sample sizes and short follow‐up periods. However, a comprehensive analysis of the available research data indicates that MSCs have broad overall therapeutic potential. Nevertheless, in the process of applying cell therapy products, it is necessary to strictly comply with a series of good manufacturing practice (GMP) guidelines formulated by the World Health Organization (WHO) and relevant health institutions [97]. These guidelines cover core contents including optimized screening methods for raw materials and standard operating procedures for cryopreservation.

On this basis, the application of MSCs in the treatment scenario of T2DM combined with OA is expected to become one of the potential directions for the treatment of such comorbidities. In addition, this review also finds that exploring the shared genes and common signaling pathways in the pathogenesis of T2DM and OA can provide brand‐new potential targets for the subsequent precise treatment of T2DM combined with OA. Future studies can further focus on the targeted regulatory effect of MSCs on the aforementioned shared genes and common pathways, and conduct targeted preclinical exploration, laying a foundation for promoting the breakthrough of treatment strategies for such comorbidities.

**Nomenclature**
AGEs: Advanced glycation end products
ACLT: Anterior cruciate ligament transection
AD‐MSCs: Adipose‐derived mesenchymal stromal cells
BM‐MSC: Bone marrow‐derived mesenchymal stromal cells
CP: Chondrocyte precursor
DFUs: Diabetic foot ulcers
DM: Diabetes mellitus
GMP: Good manufacturing practice
HA: Hyaluronic acid
haMPCs: Adipose‐derived mesenchymal progenitor cells
hMSCs: Human mesenchymal stromal cells
hUC‐MSC: Human umbilical cord mesenchymal stromal cells
IP: Intraperitoneal
KOA: Knee osteoarthritis
MetOA: Metabolic osteoarthritis
MetS: Metabolic syndrome
MSC: Mesenchymal stromal cells
OA: Osteoarthritis
PRP: Platelet rich plasma
RA: Rheumatoid arthritis
SVF: Stromal vascular fraction
STZ: Streptozotocin
T2DM: Type 2 diabetes
T2DM: Type 2 diabetes mellitus
T2DM‐OA: Type 2 diabetes in combination with osteoarthritis
TKA: Total knee arthroplasty
UC‐MSC: Umbilical cord mesenchymal stromal cells
WHO: World Health Organization
YAP1: Hippo‐yes‐associated protein.

## Ethics Statement

The authors have nothing to report.

## Disclosure

The review was written after a thorough search of the literature using the terms “Type 2 Diabetes Mellitus,“ “Diabetes Mellitus,“ “Osteoarthritis”, “Mesenchymal Stromal Cells”, and “Metabolic Osteoarthritis” in the PubMed database. Only articles written in English were included. All authors have read and approved the final manuscript.

## Conflicts of Interest

The authors declare no conflicts of interest.

## Author Contributions

Shuxing Cao, Meiling Liu, and Yongzhou Song conceived and designed the study. Material preparation, data collection, and figure creation were performed by Meiling Liu and Siyi Xie. Literature review and structural design were performed by Meiling Liu, Yajie Kong, Yiming Yang, Ruixue Chen, and Yuzhong Wang. The first draft of the manuscript was written by Meiling Liu. Meiling Liu, Yuzhong Wang, and Siyi Xie revised the final draft.

## Funding

The work was supported by grants from the Hebei Province Key R&D Plan Project (Grant 22377752D), the Medical Science Research Project of Hebei Province (Grant 20230486), the Scientific Research Fund of the Second Hospital of Hebei Medical University (Grant 2HC202210), and the Hebei Provincial Medical Research Project (Grant 20210934).

## Data Availability

All required data and code are included in text. Any further information required is available with the corresponding author.
